# Assessing childhood maltreatment on the population level in Germany: findings and methodological challenges

**DOI:** 10.1186/s13034-016-0104-9

**Published:** 2016-06-14

**Authors:** Heide Glaesmer

**Affiliations:** Department of Medical Psychology and Medical Sociology, University of Leipzig, Philipp-Rosenthal-Str. 55, 04103 Leipzig, Germany

**Keywords:** Childhood maltreatment, CTQ, General population, Childhood, Abuse, Neglect, Germany

## Abstract

Childhood maltreatment (CM) is both prevalent and consequential. Unfortunately little is known about the true prevalence of CM in the general population in Germany. The differences between findings from top down vs. bottom up approaches and the problem of the dark field of CM is discussed. Different assessment methods like trauma lists, the Childhood Trauma Questionnaire (CTQ) and the Childhood Trauma Screener (CTS) are described and the respective findings about the prevalence of CM in the adult German general population are discussed. With the example of childhood sexual abuse (SA) the challenges of quantification of CM is shown up. For instance, even if all the prevalence findings were based on methodologically sound large-scale studies, it could only be assumed that the retrospectively investigated prevalence of SA in the German general population ranges between 1.0 and 12.6 % in different studies. These findings provide an insight into the complexity of the quantification of the true prevalence of CM on the population level. Hopefully it reminds the readers of handling prevalence rates of CM carefully and to dip into the methodology of the studies before citing the respective prevalence of CM.

## Background

Childhood maltreatment (CM) is defined as “any act of commission or omission by a parent or other caregiver that results in harm, potential harm, or threat of harm to a child. Harm does not need to be intended” [1]. Hence, CM includes physical, sexual and emotional abuse as well as physical and emotional neglect (see Table 1 in [1]). CM is both prevalent and consequential and remains a major public health and social welfare problem in high income countries [1–3]. According to Gilbert et al. [1, 3] about 4–16 % of children are physically abused and around 10 % of children are neglected or psychologically abused [1]. CM substantially contributes to child mortality and morbidity. The long-lasting effects on mental and physical health, substance abuse, risky sexual behaviour, and criminal behaviour persist into adulthood [1, 2, 4]. Due to its prevalence as well as its complex and cumulative effects on the developing brain, mind and body CM is perhaps one of the most important factors to assess in a variety of contexts [5]. Additionally detection and reporting of CM matters to promote child safety and health and to inform professionals in health care, in educational and law system as well as policy makers [3]. Drawing on the example of the assessment of CM on the population level in Germany and especially of sexual abuse (SA), the challenges and pitfalls of the assessment of CM, will be discussed in the following.

### Assessment of CM

Essentially, there are two approaches of quantification of CM on the population level: a top down and a bottom up approach. While the top down approach uses official statistics from child protection agencies or reports to the police, the bottom up approach uses data from epidemiological studies in different populations like children of different ages, adolescents and adults. The prevalence of CM from a bottom up assessment is much higher than from top down sources. This provides strong evidence that a larger proportion of CM is not reported [3]. This underrecognized and underreported share of CM is called the “dark field of childhood maltreatment”. To light this dark field is one of the major challenges. A combination of evidence from both approaches and all available sources seems promising for the estimation of the true prevalence of CM.

Several well-established instruments for the assessment of CM in clinical and epidemiological research are available to date. The spectrum ranges from self-report measures to (standardized) interviews, and from categorial (yes vs. no; e.g. list of traumatic events) to dimensional measures of CM. A recent systematic review gives an insight into the usually applied assessment methods in population surveys [6]. In large-scale epidemiological studies economic assessment tools are needed to support feasibility of the study protocols. Thus complex and comprehensive measures are not always the usual assessment tools applied in population surveys [6].

The most economic assessment is the use of self-report lists of traumatic events, e.g. *Traumalist of the M-CIDI* [7]. These lists usually have a dichotomous format, hence the participants indicate whether they have experienced different kinds of traumatic events or not. This forthright way of assessment requires participants capable of memorizing and critically reflecting upon their experiences  as well as a kind of precise phenomenological understanding of a specific traumatic event (e.g. what exactly means sexual abuse). Thus such lists might be suitable for the assessment of commonly defined traumatic events like car accident or natural disaster. However the assessment of emotional neglect or sexual abuse might not work well with a traumalist. Moreover this specific type of list does not allow assessing frequency, duration and severity of the respective experiences and requires self-identification of the respondents.

The *Childhood Trauma Questionnaire* (CTQ) [8] is an internationally established tool for the retrospective assessment of CM in adolescent and adult populations [9]. The original version of the CTQ was developed from a 70-item questionnaire. In further studies the questionnaire was reduced to a 28-item version using exploratory and confirmatory factor analyses. This 28-item questionnaire is the most commonly used version applied in a vast number of studies in different languages and settings. Based on theoretical assumptions the CTQ consists of five subdimensions: physical abuse (PA; e.g. “…got hit so hard that I had to see a doctor or go to the hospital”), sexual abuse (SA, e.g. “…someone tried to touch me in a sexual way/made me touch him.”), emotional abuse (EA, e.g. “…people in my family called me stupid, lazy or ugly.”), physical neglect (PN, e.g. “…I knew there was someone to take care of me and protect me.”), and emotional neglect (EN, e.g. “…someone in my family helped me feel important or special.”, reverse coded) with five items representing each subdimension with a five-point likert scale for each item (1 = “never” to 5 = “very often”). The sum of the five items for each subscale ranges from 5 to 25. According to the original manual the sumscores of the subscales are classified for severity on four levels [8]. A slightly different procedure of severity ratings was recommended by Walker et al. [10] with a dichotomous differentiation of CM. These cut-off criteria had been ascertained by relating CTQ subscale scores to ratings of expert blinds for the CTQ scores who administered detailed clinical interviews. Based on the fulfillment of consensus childhood abuse and neglect criteria, experts determined whether participants had a history of clinically significant abuse or neglect [10]. Table 1 gives an overview about both scorings. According to Walkers approach PA and PN include all cases from “slight to moderate” up to “extreme” CM, SA and EN include all cases from “moderate to severe” up to “extreme” CM. For EA the cut-off is in the middle of the “slight to moderate”-level.

**Table 1:** Classification of abuse and neglect along the sum scores of the subscales

	Classification according to Bernstein [8]				Classification according to Walker [10]
	None to minimal	Slight to moderate	Moderate to severe	Severe to extreme	
Emotional abuse	5–8	9–12	13–15	16–25	10–25
Physical abuse	5–7	8–9	10–12	13–25	8–25
Sexual abuse	5	6–7	8–12	13–25	8–25
Emotional neglect	5–9	10–14	15–17	18–25	15–25
Physical neglect	5–7	8–9	10–12	13–25	8–25

There is mixed evidence about the dimensionality of the CTQ, with some indications that its structure may vary across different groups. Especially the psychometric properties of the PN subscale are subject to a critical debate [8, 11–14]. The internal consistencies of the subscales lay between 0.62 and 0.96 [8]. As a measure of test–retest reliability at a median interval of 6 weeks, the intraclass coefficient were 0.77 for the CTQ as a whole and 0.58–0.81 for the subscales [15]. The results of the CTQ show moderate correlations with those of semistructured interviews (from 0.43 for physical and emotional abuse to 0.57 for sexual abuse) [16]. Furthermore, the results of the CTQ show correlations with ratings by psychotherapists from 0.42 for physical neglect to 0.72 for sexual abuse [17].

Despite the fact that some evidence suggests moderate to good consistency of self-reports of maltreatment over time, the retrospective nature of the CTQ carries some risk of response bias that could possibly undermine the validity of this instrument. Hence, besides the 25 items representing five subscales of the CTQ another 3-item-response-bias scale called minimization-denial scale (MD) was included by the original authors. Unfortunately, the overwhelming majority of studies reporting CTQ data neither include information about MD items nor take these items into account for analyses and interpretation [18]. Thus little is known about this MD measure. Moreover, if response biases are common and consequential, current practices of minimizing the MD scale deserve revision. Thus, a recent re-analysis of data from 24 multinational samples with a total of 19,652 participants was performed [19]. Overall, results of this analysis suggest that a minimizing response bias—as detected by the MD subscale—has a small but significant moderating effect on the discriminative validity of the CTQ. Researchers and clinicians should be cautioned about the widespread practice of using the CTQ without the MD scale, or collecting MD data but failing to control for its effects on outcomes or dependent variables [19].

To support the economic assessment CM a short screening instrument was developed based on the German version of the CTQ. The *Childhood Trauma Screener* (CTS) consists of 5 items (each item representing one subscale of the CTQ [20]. The correlations between the 5 items and the respective subscales of the CTQ range between r = 0.55 and r = 0.87. Internal consistency of the CTS was good (α = 0.757) [20]. To support the application of the CTS for categorical diagnostics cut-offs of the different dimensions of CM have been defined based on two large-scale population studies in Germany [21]. A further investigation of psychometric properties of the CTS is necessary.

### CM on the population level in Germany

The findings from several studies investigating CM on the population level in Germany are outlined and discussed below. Table 2 gives an overview about the core methodological characteristics of the different studies. Frequency and severity of CM in the adult German population was investigated using the CTQ in a population-based representative study in 2010 [22]. The data have already been published. For more detailed information please refer to the original publications [22, 23]. Table 3 gives an overview about the frequency of CM according to the four severity levels recommended by Bernstein [8, 23] and according to the dichotomous approach recommended by Walker [10, 22] from this study. The application of different cut-offs for the definition of caseness leads to different statements about the frequency of CM on the population level (Table 3).

**Table 2:** Methodological characteristics of the population studies discussed in the paper

	Population-based representative study 2005	Population-based representative study 2007	Population-based representative study 2010	SHIP-Legende 2007–2010
Area covered by the study	Population-based representative study for Germany	Population-based representative study for Germany	Population-based representative study for Germany	Population-based study in the northeastern part of Germany (Pomerania)
Sample size	2426	2510	2504	2400
Response rate	60.9 %	61.9 %	56 %	Of the n = 4308 participants at SHIP-baseline, n = 3669 were invited for SHIP-Legende. Of those 92 died between 2007 and 2010, 1011 refused participation, 132 were not reached and 35 did not attend the assessments
Age range (years)	14–93	14–92	14–90	29–89
% Female participants	53.9	54.5	53.2	52.4
Mode of assessment	All subjects were visited by a study assistant, informed about the investigation, and self-rating questionnaires were presented. Assistant waited until participants answered all questionnaires and offered help if persons did not understand the meaning of questions	All subjects were visited by a study assistant, informed about the investigation, and self-rating questionnaires were presented. Assistant waited until participants answered all questionnaires and offered help if persons did not understand the meaning of questions	All subjects were visited by a study assistant at home, informed about the investigation, and self-rating questionnaires were presented. Assistant waited until participants answered all questionnaires and offered help if persons did not understand the meaning of questions	All subjects were supported by a study assistant, informed about the investigation, and self-rating questionnaires were presented in the private homes or in one of the both SHIP-study centers. The assistants offered help if persons did not understand the meaning of questions
Instruments assessing CM	Trauma-list (M-CIDI)	Trauma-list (M-CIDI)	CTQ/CTS	CTS
Related publications	[25]	[24]	[21–23]	[21]
Funding	University of Leipzig	University of Leipzig	University of Leipzig	German Research Foundation
	Department of Medical Psychology and Medical Sociology	Department of Medical Psychology and Medical Sociology	Department of Medical Psychology and Medical Sociology

**Table 3:** Frequency and severity of CM in the German general population

	CTQ—classification according to Bernstein [8]^a^								CTQ—classification according to Walker [10]^b^		CTS German community sample (SHIP LEGENDE)^c^		CTS Representative German sample 2010^c^	
	None to minimal		Slight to moderate		Moderate to severe		Severe to extreme							
	n	%	n	%	n	%	n	%	n	%	n	%	n	%
Emotional abuse	2123	84.8	259	10.3	75	3.0	40	1.6	254	10.2	110	5.2	170	6.7
Physical abuse	2198	87.8	162	6.5	70	2.8	69	2.7	301	12.0	99	4.7	132	5.3
Sexual abuse	2186	87.3	158	6.3	109	4.3	47	1.9	156	6.2	92	4.3	172	6.9
Emotional neglect	1259	50.3	888	35.5	184	7.3	164	6.5	348	13.9	214	10.1	167	6.7
Physical neglect	1288	51.4	491	19.6	450	18.0	269	10.8	1210	48.4	226	10.6	364	14.7

^a^Published data, for more details see [23]

^b^Published data, for more details see [22]

^c^Published data, for more details see [21]

The CTS as a short screening tool out of the CTQ was used in two samples to quantify the frequency of CM [21]. One study is a large-scale community sample (Study of Health in Pomerania) from northeastern Germany the other one is the population-based representative sample mentioned above (for more details see Table 2). The prevalences of CM from both studies are presented in Table 3. The results differ slightly in both samples. Currently it is impossible to determine whether this is attributable to the differences in both samples (population-based representative German sample vs. community sample from northeast of Germany, see Table 2) or to the psychometric problems of a short screener, such as the CTS. Further research is needed to verify the psychometric properties of the CTS.

Additionally, in 2005 and 2007 two population based representative surveys assessed the frequency of traumatic events in Germany, including childhood sexual abuse (up to the age of 14), using a traumalist [24, 25] (for more details concerning methodology see Table 2). The findings of both studies are comparable with a prevalence of childhood sexual abuse of 1.2 % in the study of 2005 [25] and 1.0 % in the study of 2007 [24].

## Conclusions

The prevalence of CM in the general population in Germany assessed with a bottom up approach depends on the instrument used and the applied cut-off scores. The example of experiences of childhood sexual abuse in the German general population, illustrates what this means. Using a trauma list (with a dichotomous answer format) the prevalence of SA ranges between 1.0 and 1.2 % [24, 25]. Using the CTQ as a dimensional self-report measure with five subscales, the prevalence of SA is 6.2 vs. 12.6 % depending on the cut-off-score. Based on the CTS the prevalence of SA is 4.3 vs. 9.5 % in two different samples (for details see Table 2). With this example of childhood sexual abuse the challenges of the quantification of CM is shown up. Even if all these prevalence data are based on methodologically sound large-scale studies, we can only say that the retrospectively investigated prevalence of SA in the German adult population ranges between 1.0 and 12.6 %.

There are several sources of error: (1) representativeness of the population under study; (2) recall bias, especially for retrospective measures like the CTQ; (3) the quality of the assessment instrument. The studies discussed above are large-scale population based samples which are methodically sound with respect to representativeness, sample size etc., Nevertheless they were assessing CM retrospectively and especially in the older age groups these studies refer to experiences decades ago. Thus a critical reflection about recall bias is important. From a psychometric or methodological perspective, dimensional measures with several items assessing every subdomain of CM including a rating of the frequency of the experiences (e.g. CTQ) seem to be more reliable measures than a dichotomous item on a trauma list. Hence, with the use of dimensional measures the question of the correct cut-off-score arises. The big question is: Can we recommend one cut-off-score for the CTQ, in different settings (clinical vs. general population), different cultural backgrounds or different age-groups? Even if this is not an easy to handle recommendation it seems worthwhile to discuss different cut-off-scores depending on the field of application (e.g. lower cut-offs for screening). Moreover, the length of an instrument and its operationalization is a very important topic and a possible source of error. For instance the CTQ-subscale PN includes one item “I didn’t have enough to eat.” This item is a possible source of error when applied in the German elderly who grew up in the postwar-period in Germany with very common experiences of shortages of food etc. in this time. Thus this item will lead to an overestimation of PN in this age group. Additionally, the items of the CTQ are more or less clear, e.g. “I got hit so hard by someone in my family that I had to see a doctor or go to the hospital.” is operationalizing PA in a behavioural manner. On the other hand, an item like “I felt loved.” assesses the feeling of being loved with some aspect of interpretation what that could mean and carries a margin for interpretation. Even though the problem of fixing the prevalence of CM in the general population in Germany is not resolved with all these studies, this compilation of data from Germany gives an insight in the complexity of the problem. Hopefully, it reminds the readers in handling prevalence information about CM with care and to dip into the methodology of the studies before citing prevalence rates of CM.

## Abbreviations

CM: childhood maltreatment
PN: physical neglect
EN: emotional neglect
PA: physical abuse
EA: emotional abuse
SA: sexual abuse
CTQ: Childhood Trauma Questionnaire
MD: minimization-denial scale
CTS: Childhood Trauma Screener
M-CIDI: Munich Composite International Diagnostic Interview
